# A Deep Machine Learning Method for Concurrent and Interleaved Human Activity Recognition

**DOI:** 10.3390/s20205770

**Published:** 2020-10-12

**Authors:** Keshav Thapa, Zubaer Md. Abdullah Al, Barsha Lamichhane, Sung-Hyun Yang

**Affiliations:** Department of Electronics Engineering, Kwangwoon University, Seoul 139-701, Korea; kshavthapa@kw.ac.kr (K.T.); abdullah@kw.ac.kr (Z.M.A.A.); barshaer@kw.ac.kr (B.L.)

**Keywords:** concurrent, interleaved, RNN, BiLSTM, SCCRF, activity recognition, Smart Home

## Abstract

Human activity recognition has become an important research topic within the field of pervasive computing, ambient assistive living (AAL), robotics, health-care monitoring, and many more. Techniques for recognizing simple and single activities are typical for now, but recognizing complex activities such as concurrent and interleaving activity is still a major challenging issue. In this paper, we propose a two-phase hybrid deep machine learning approach using bi-directional Long-Short Term Memory (BiLSTM) and Skip-Chain Conditional random field (SCCRF) to recognize the complex activity. BiLSTM is a sequential generative deep learning inherited from Recurrent Neural Network (RNN). SCCRFs is a distinctive feature of conditional random field (CRF) that can represent long term dependencies. In the first phase of the proposed approach, we recognized the concurrent activities using the BiLSTM technique, and in the second phase, SCCRF identifies the interleaved activity. Accuracy of the proposed framework against the counterpart state-of-art methods using the publicly available datasets in a smart home environment is analyzed. Our experiment’s result surpasses the previously proposed approaches with an average accuracy of more than 93%.

## 1. Introduction

**Figure 1:**
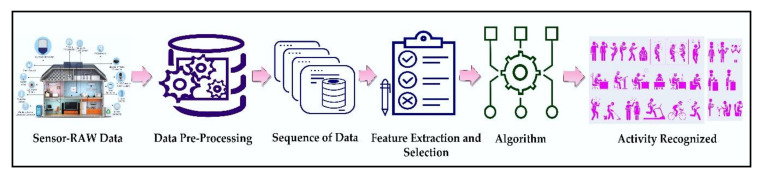
General Process for Activity Recognition.

Human activity recognition has been a foremost research field since last decades due to advancements in technologies and its applicability in different domains like home automation, elderly care [1], telemedicine services [2], ubiquitous computing [3], and so forth. Among them, Smart homes embedded with many most uncomplicated, portable, fastest sensors have gained much attention in the field of ambient assisted living for prospering the quality of resident’s life [4,5]. Based on the sensors used, wearable sensors and external sensors are used to identify the activity. The wearable sensor attached to the human body measure the signal patterns obtained through a movement of the human body. External sensors installed into different locations of the environment helps to recognized activity according to its generated value. One importance of activity recognition is to provide information on a human’s behavior that allows computing systems to assist them in their daily tasks proactively. However, recognizing activities is a challenging task because human activities are complex and uncertain. Thus, only a few approaches address complex activity recognition [6]. Many of these approaches detect single user-single activity at a time, and a consecutive sequence of sensors data can achieve that. Nevertheless, multiple-user multiple-activity exists within a single sequence of actions as a concurrent and interleaving activity [7]. The recognizing of two activities concurrently through an observed activity sequence is called concurrent activity. Likewise, if one activity is paused (store for a short time) and then resumes after executing another activity is called interleaved activity. Recognition of activity mainly consists of three steps, as shown in Figure 1. First, the sliding window technique is applied to segments a stream of raw sensor data. More of all window shifts a fixed time-length or a set number of sensor data along the stream with overlapping or non-overlapping between adjacent segments. In the second step, we extract features (e.g., max, min, mean, variance, and entropy) from each segment and represent the feature vector’s raw signals. The last step is called training and testing [8,9,10]. Recognition of activities needed detailed user specification, a large amount of training data, sophisticated network, and time-varying sequential data processing. The machine learning method solves these kinds of problems.

Necessarily, many different probabilistic and non-probabilistic machine learning methods [11,12,13] have been hot-figure for activity recognition in recent years. However, difficulties confronted by traditional machine learning approaches are overcome by deep learning and led to numerous enhancement in activity recognition. A deep learning method called LSTM, A recurrent neural network, is used to recognize published by [14]. These techniques have already better performed than other machine learning algorithms in many applications like speech recognition [15], text recognition [16], image recognition [17] computer vision, including human activity recognition. The skip chain CRF [18] established the correlation between non-consecutive identical activities in the environment. This skip-chain is a probabilistic model; it has an advantage in recognizing activity naturally and conveniently. Therefore this unique feature of learning sequential, feedback connections, and storage ability of LSTM and recognizing identical features or data along long edges of SCCRF makes them recognize the composite activities. In this paper, we introduce the approach that contains a single platform by combining the features of BiLSTM and SCCRF to recognize these complex activities. This proposed approach could have been the first proposed so far to date. Our proposed method highlights;

- We present the two-phase recognition method. The first phase is capable of automatically learning concurrent feature representations and modeling the temporal dependencies between their activation to detect the concurrent activity with a deep learning framework composed of Bi-directional LSTM. The second phase explicitly models dependencies between distant activity and turns out to be particularly useful in interleaved activity detection using SCCRF.
- A feature-learning structure can directly learn spatial-temporal features from the raw data via LSTM structures, which requires neither manual feature selection nor classifier selection.
- The proposed framework can be applied seamlessly to different recognition modalities and other recognition platforms.
- The system adopts the raw sensor data with less preprocessing, which makes it exceptionally comfortable and general.
- We compare the performance of our framework to publically available datasets from Kasteren and Kyoto (WSU).
- The results depicted by our proposed framework outperforms published results on recognition of concurrent and interleaved activity.
- The proposed approach can classify the variable window ranges of human activities. Utilizing the LSTM to read variable window ranges, sequences of input sensors data can later recognize the entire window segment’s activity.

The remainder of the paper is organized as follows; related work is in Section 2. Section 3 illustrates the materials and method related to the proposed approach. Part 4 is about our experimental configuration for the activity recognition method. The activity recognition performance analysis is described in Section 5. Section 6 describes a conclusion.

## 2. Related Work

Activity recognition aims to recognize a user’s high-level activity from different sensors or actions the user performed. In this case, they mostly like to have either logical-based approaches or probabilistic approaches. [19] Explained the logic-based approach, which provides a formal theory of activity recognition. Still, these approaches have limited inconsistent activity, unable to handle uncertainty, single activity recognition, maximum noise appearance, and significant rule of conduct. A procedure tries to solve the multiple-activity recognition problem in [20]. In their approach, a finite state machine creates transitions between different activity states, i.e., interleaving activity. However, this also fails to handle uncertainty. Concurrent activity recognition using a single classifier, encoding a binary output vector, has gained little attention, but its timing problem overruled activity’s concurrency.

In the millennium, following the success of machine learning [21] and deep learning [22], wearable sensor-based activity recognition came in noticed. Restricted Boltzmann machine RBM on deep learning methods has not significantly improved deep belief network (DBNs) [23] with multiple layers. The Hidden Markov models also have been exploited above RBM layers on DBN based models harness temporal sequences in human activities. However, HMM performs well on limited numbers of hidden states and trying to modeling on long dependencies.

Human Activity Recognition with Convolutional Neural Networks (CNN) [24], recurrent neural network RNN [25], and other deep learning techniques still use wearable sensors or body-worn sensors and can only detect single and straightforward activity. Whoever detects some complex activity suffers from accuracy and complexity. If they use external sensors also, they only focus on just the single activity recognition. Many researchers also used classification algorithms such as skip chain conditional random field (SCCRF) [7], Decision Tree [26], Hidden Markov Model (HMM) [27], Conditional Random Field (CRF) [28]. Still, these approaches get rid of temporal differences between events. In this work, we focus most of our analysis on concurrent and interleaving activity using the extended LSTM and CRF due to their widespread popularity and a proven track record. The growing interest in LSTM-RNN enhanced many technical applications such as language modeling, handwriting recognition, and generation [29], machine translation [30], speech recognition [31], video analysis [32], and image captioning [33] least compare to activity recognition. SCCRF were also have been used for natural language processing and other specific task detection problem like behavior recognition [34]. Meanwhile, an abundance of work, architecture, and model has been developed, but relatively less attention has been paid to understand the properties of its concurrency and interleaved representations and predictions.

## 3. Materials and Method

**Figure 2:**
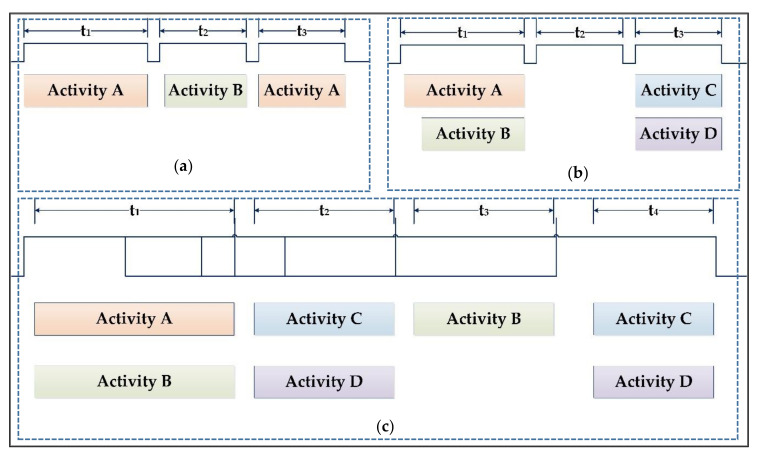
Pictorial view of (**a**) Interleaved Activity occurrence, (**b**) Concurrent Activity occurrence, (**c**) Concurrent and Interleaved Activity.

Figure 2 explains the overall system (process) of activity recognition. While recognizing activity phenomena, our interest is in recognizing complex activity such as concurrent and interleaved activity. The activities done at the same time is a concurrent activity, such as talking with friends while walking or listening to music while sleeping. Both the activities must be recognized concurrently, as shown in Figure 2a. When one activity is paused to do another activity and continues the previous activity after finishing, that is the interleaved activity. That means some real-life activities are interleaved in nature, as shown in Figure 2b. For instance, if a bell rings at the door while watching TV, we pause watching TV and go to open the door, and we continue to watch TV. The activities always may not be concurrent and interleaved separately; it may occur in the combine, as shown in Figure 2c. Sleeping by listening to music can be paused while talking with friends on the phone and may continue sleeping by listening to music after talking is over. This paradigm is the most critical aspect of activity recognition but its always been avoid due to its complexity.

### 3.1. Long-Short Term Memory (LSTM)

**Figure 3:**
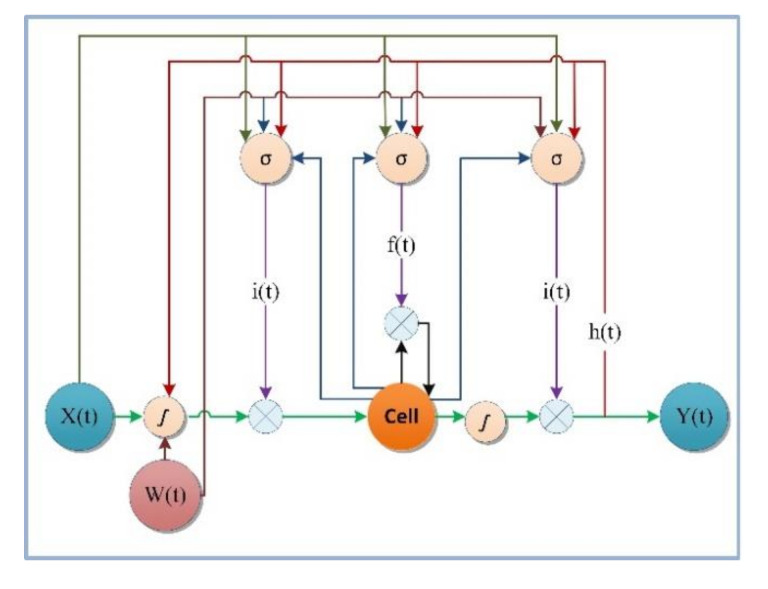
LSTM Architecture.

Recurrent Neural Networks (RNNs) make the output use the previously memorized information extracted from the past data to the neurons. This specific ability of RNNs of the neurons to find patterns with long-term dependencies. However, the problem of vanishing gradient may appear. These happened when the error function derivatives concerning the network weights become either very large or close to zero during the training phase effects the RNNs. The proposed Long-Short-Term-Memory (LSTM) cell addresses this issue. LSTM designed to memorize the information over time by storing it in an internal memory called a cell and update output or erase their internal state depending on their input and the state at the previous time step. LSTM networks capture temporal dependencies in diversified application fields such as image captioning, automatic translation, or video-based activity recognition. The LSTM architecture comprises memory cells with self-connections for storing high temporal dependencies, as shown in Figure 3. They contain the gates to control the flow of information. The memory cell consists of a node with a fixed weight with a self-connect loop edge so that the gradient can propagate across many time steps without vanishing. An input gate controls the input activation data into memory cells; the primary function of this gate is to write on memory cells. An output gate control and read output data flow of cell activations. Forget gate decides which data to be forgotten or resetting the cell’s memory.

### 3.2. Bi-Directional LSTM

**Figure 4:**
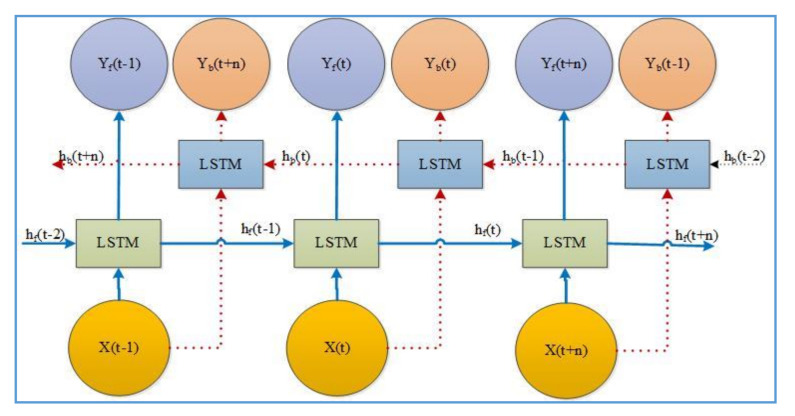
Bi-directional LSTM architecture.

The baseline LSTM algorithm can recognize current activity based only on prior information. However, some vital information about the activity may not be adequately extracted by the cell if it processes only one direction. The principal feature in bidirectional LSTM is that it follows information and current output related to former details. Bidirectional LSTM is a design of two LSTM cells, and the output is determined together, as shown in Figure 4.

The LSTM architecture also contains peephole in internal cells to gates in the same cell to learn the outputs’ precise timing. The multiplicative equation controls each cell and gate to the forward propagation:

(1) $$i\left(t\right)=\sigma \left(w_{xi}x\left(t\right)+w_{hi}h_{f}\left(t-1\right)+w_{ci}c\left(t-1\right)+b_{i}\right)$$

(2) $$f\left(t\right)=\sigma \left(w_{xf}x\left(t\right)+w_{hf}h_{f}\left(t-1\right)+w_{cf}c\left(t-1\right)+b_{f}\right)$$

(3) $$o\left(t\right)=\sigma \left(w_{xo}x\left(t\right)+w_{ho}h_{f}\left(t-1\right)+w_{co}c\left(t-1\right)+b_{o}\right)$$

(4) $$c\left(t\right)=f_{t}c\left(t-1\right)+i\left(t\right)tanh_{f}\left(w_{xc}x\left(t\right)+w_{hc}h\left(t-1\right)+b_{c}\right)$$

(5) $$h_{f}\left(t\right)=o\left(t\right)tanh_{f}c\left(t\right)$$

$x\left(t\right)$ is the input sequence and $w_{xi}$, $w_{xf}$ and $w_{xo}$ being the input weight matrix of $i\left(t\right)$ an input gate, $f\left(t\right)$ a forget gate and $o\left(t\right)$ an output gate, having the same vector size as the vector of hidden value.

The c represents the memory cell, and b represents the bias of the respective gate and cell. The hidden state $h\left(t\right)$ processes input $x\left(t\right)$ and thus yields output $y\left(t\right)$ at time t by unrolling LSTM cells, as shown in Figure 4. σ; known as the logistic sigmoid function or the sigmoid activation function that limits the value in between [0,1] and given as $\sigma \left(x\right)=\frac{1}{1+e^{-x}}$. tanh; Hyperbolic tangent activation function. The forward propagation executes input x (t) from left to right with a hidden layer of $h_{f}$, and backward propagation executes input from right to left with the hidden layer of $h_{b}$ as according:

(6) $$\delta_{i}\left(t\right)=\sigma \left(w_{yi}x\left(t\right)+w_{hi}h_{b}\left(t+1\right)+w_{ci}c\left(t+1\right)+b_{i}\right)$$

(7) $$\delta_{f}\left(t\right)=\sigma \left(w_{\mathrm{yf}}x\left(t\right)+w_{\mathrm{hf}}h_{b}\left(t+1\right)+w_{\mathrm{cf}}c\left(t+1\right)+b_{f}\right)$$

(8) $$\delta_{o}\left(t\right)=\sigma \left(w_{\mathrm{yo}}x\left(t\right)+w_{\mathrm{ho}}h_{b}\left(t+1\right)+w_{\mathrm{co}}c\left(t+1\right)+b_{o}\right)$$

(9) $$\delta_{c}\left(t\right)=f_{t}\delta_{c}\left(t+1\right)+\delta_{i}\left(t\right)tanh_{b}\left(w_{yc}x\left(t\right)+w_{hc}h_{b}\left(t+1\right)+b_{c}\right)$$

(10) $$h_{b}\left(t\right)=\delta_{o}\left(t\right)tanh_{b}\delta_{c}\left(t\right)$$

Therefore, in this work, we focused on recognizing concurrent and interleaving activity and its analysis. Meanwhile, an abundance of research work modifies or extends LSTM architecture to understand the properties of its complex representations and predictions. [35] Recently evaluated a comprehensive study of LSTM components. The most leading deep LSTM networks for the HAR method for human activity recognition using wearable sensing approaches exploit the contextual information and adopt the temporal dependencies in variable-length input data [36].

### 3.3. Skip Chain CRFs

**Figure 5:**
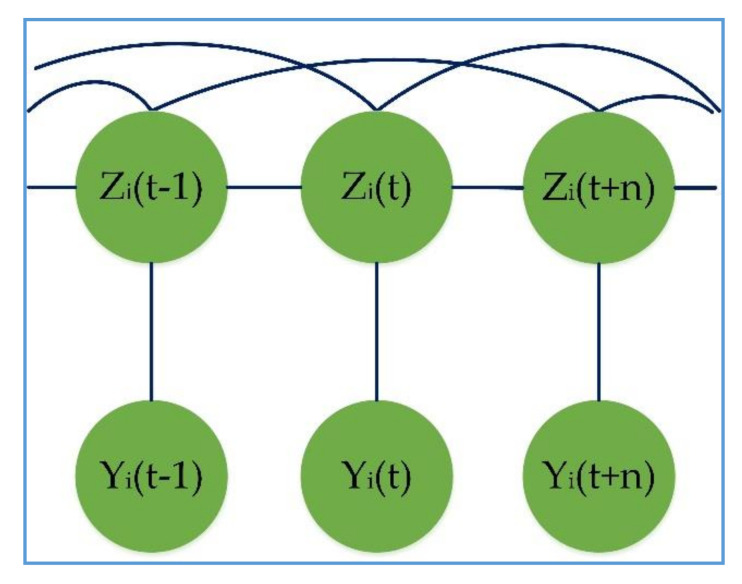
SCCRF Representation.

The SCCRFs can represent dependencies between distant nodes so that similar activity can be detected. The SCCRF [37] can contain information from the activity of both times. Because the activity of one time is specific, it can impact the activity of the other time. We have to choose an identical activity that belongs to the same class or have a small edit distance, as shown in Figure 5.

However, we connect all possible activity because it requires excessive programming algorithm. Therefore we are only concern about the edges (activity) that appeared to be active or not at time slice t. Given observation sequence Y, let Z be all pairs of active activities connected with skip edges. Thus the probability of activity sequence Z given an observation activity Y can be written as;

(11) $$P\left(z,y\right)=\frac{1}{S\left(y\right)}\overset{n}{\underset{t=1}{\prod }}\Phi (z_{t},z_{t-1},y,t)\;\underset{\left(uv\right)\in I}{\prod }\Psi (z_{u},z_{v},y,u,v)$$

Φ is the values over linear-chain edges, and Ψ is the potential factor over skip edges. S(y) is a normalization factor where each of the potential factors is factorized by taking the log.

(12) $$\Phi (z_{t},z_{t-1},y,t)=\exp\underset{k}{\sum }\lambda_{k}f_{k}(z_{t},z_{t-1},y,t)$$

(13) $$\Psi (z_{u},z_{v},y,u,v)=\exp\underset{k}{\sum }\lambda_{k}^{\prime }f_{k}^{\prime }(z_{u},z_{v},y,u,v)$$

$\lambda_{k}$ and $\lambda_{k}^{\prime }$ are the parameters of the linear and skip chain template on which each of them factorizes according to a set of features $f_{k}$ or $f_{k}^{\prime }$ respectively. In skip-chain CRFs, exact inference $P\left(z|y\right)$ is much more difficult due to long loops and overlapping in the model. Instead, we approach approximate inference utilizing the belief propagation algorithm that works as the principle of the forward-backward algorithm for HMM. However, LPA passages information between the long-distance edges, emitting probabilistic information from distant sequences.

For the given training data, we estimate the parameter $\lambda_{k}$ by maximizing the log-likelihood after calculating the partial derivative and optimization technique

(14) $$\frac{\partial P\left(z|y\right)}{\partial \lambda_{k}}=\frac{\partial L}{\partial \lambda_{k}}=\frac{\lambda_{k}}{\sigma^{2}}$$

where L log-likelihood and $\sigma^{2}$ is the covariance as assuming that the long-distance parameter is similar, therefore replacing $\lambda_{k}^{\prime }$ by $\lambda_{k}$ and $f_{k}^{\prime }$ by $f_{k}$.

### 3.4. Proposed Method

**Figure 6:**
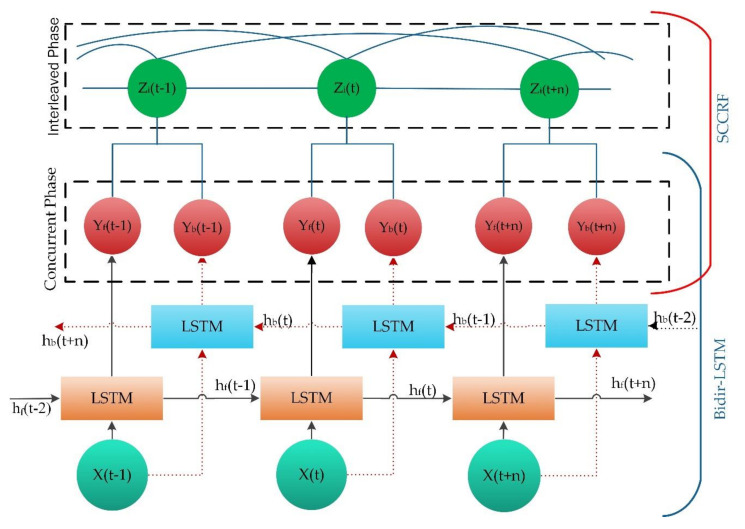
Proposed schematic architecture.

**Figure 7:**
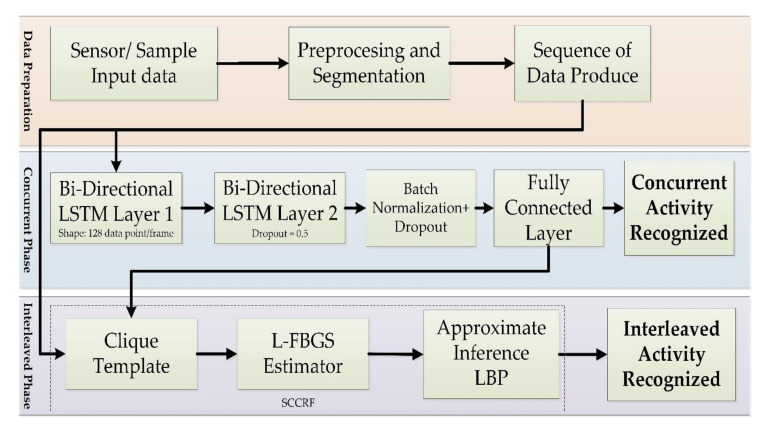
Overall system structure of the proposed approach.

The schematic architecture in Figure 6 demonstrates complex activity recognition. Our proposed approach has the two-phase structure in which the first phase processes under the bi-directional LSTM theorem, and the second phase implement a theory of Skip-Chain CRF (SCCRF). It performs a direct mapping from raw multi-modal sensor inputs to activity classification and recognition. It recognizes the label of performed activity during a specific time window. The input is an equally spaced discrete sequence of samples ($x_{t-2},x_{t-1}x_{t},x_{t+1}\ldots ,x_{t+n}$) where each data point $x_{t}$, is a set of individual observed samples by sensors at time t. The extracted samples are segmented into windows and fed to the BLSTM-based model. The model outputs a sequence of scores representing activity predictions. The first phase represents the detection of concurrent activity; second, interleaved activity. The bi-direction LSTM network contains two parallel LSTM tracks; forward and backward propagations for exploiting data from past and future of a specific time step to recognize the parallel activity. The output of the bi-directional LSTM determines the concurrency of the recognized activity. The same output is feedforward to recognize an interleaved condition of activity using the Skip-Chain Conditional Random Field (SCCRF). The model outputs a sequence of activity ($y_{t-2},y_{t-1}y_{t},y_{t+1}\ldots ,y_{t+n}$) in which there is an activity prediction for each time step ($z_{t-2},z_{t-1},z_{t},z_{t+1}\ldots ,z_{t+n}$). SCCRF finds the probability of activity that has been paused to perform a crucial uncertain task. The SCCRF is very reliable and efficient in finding the possibility of a similar activity performed in activity sequences. A schematic diagram in Figure 7 gives concepts of an overall system architecture of the proposed. In the first phase, the input sensor data or sampled data is transformed through a sliding window to produce data; this procedure is regarded as data preprocessing. Prepared data is inserted into the first layer Bi-LSTM is processed until it adapts its internal state. An passed into another Bi-LSTM layer where we applied the dropout to drop the unused neurons. The obtained result is normalized and fed into a fully connected layer, which is also known as the coding layer and hence recognizes the concurrent activity. In the second phase, the data’s sequence and the data from the fully connected layer are inserted into the clique template. Clique template makes surmise on the structure of data by defining the configuration of the cliques. A clique is a set of interdependent data. And finally, the SCCRF model is used to recognize the interleaved activity. Our concern target is to develop a reliable algorithm implementing both the machine learning and deep learning into a single frame (Algorithm 1).

**Algorithm 1** pseudocode for the proposed algorithm1. initialize network 2. reset : inputs = 0, Activations = 0      forward propagation: 3. initialize the inputs do 4. roll over: Activations; cell states 5. loop over a cell, end for 6. do   for t=0 to n do     Calculates the gate values :         inputs gates: $i\left(t\right)\leftarrow \sigma \left(w_{\mathrm{xi}}x\left(t\right)+w_{\mathrm{hi}}h_{f}\left(t-1\right)h_{f}\left(t-2\right)+w_{\mathrm{ci}}c\left(t-1\right)+b_{i}\right)$
        forget gates: $f\left(t\right)\leftarrow \sigma \left(w_{\mathrm{xf}}x\left(t\right)+w_{\mathrm{hf}}h_{f}\left(t-1\right)h_{f}\left(t-2\right)+w_{\mathrm{cf}}c\left(t-1\right)+b_{f}\right)$
        loop over the cells is block now         output gates: $o\left(t\right)\leftarrow \sigma \left(w_{\mathrm{xo}}x\left(t\right)+w_{\mathrm{ho}}h_{f}\left(t-1\right)h_{f}\left(t-2\right)+w_{\mathrm{co}}c\left(t-1\right)+b_{o}\right)$
        update the cell:$c\left(t\right)\leftarrow f_{t}c\left(t-1\right)+i\left(t\right){\tan h}_{f}\left(w_{\mathrm{xc}}x\left(t\right)+w_{\mathrm{hc}}h_{f}\left(t-1\right)h_{f}\left(t-2\right)+b_{c}\right)$
          final hidden state/ final output : $h_{f}\left(t\right)\leftarrow o\left(t\right)⊙\tanh_{f}c\left(t\right)$
   end for 7. Single activity detect 8.  do   Update the weight   end for 9. backward propagation do       for t=0 to n       inputs gates: $\delta_{i}\left(t\right)\leftarrow \sigma \left(w_{\mathrm{yi}}x\left(t\right)+w_{\mathrm{hi}}h_{b}\left(t+1\right)h_{b}\left(t+2\right)+w_{\mathrm{ci}}c\left(t+1\right)+b_{i}\right)$
    forget gates: $\delta_{f}\left(t\right)\leftarrow \sigma \left(w_{\mathrm{yf}}x\left(t\right)+w_{\mathrm{hf}}h_{b}\left(t+1\right)h_{b}\left(t+2\right)+w_{\mathrm{cf}}c\left(t+1\right)+b_{f}\right)$
    output gates: $\delta_{o}\left(t\right)\leftarrow \sigma \left(w_{\mathrm{yo}}x\left(t\right)+w_{\mathrm{ho}}h_{b}\left(t+1\right)h_{b}\left(t+2\right)+w_{\mathrm{co}}c\left(t+1\right)+b_{o}\right)$
    cell output: $\delta_{c}\left(t\right)\leftarrow f_{t}\delta_{c}\left(t+1\right)+\delta_{i}\left(t\right){\tan h}_{b}\left(w_{\mathrm{yc}}x\left(t\right)+w_{\mathrm{hc}}h_{b}\left(t+1\right)h_{b}\left(t+2\right)+b_{c}\right)$
10. Hidden state / final output : $h_{b}\left(t\right)\leftarrow \delta_{o}\left(t\right)⊙{\tan h}_{b}\delta_{c}\left(t\right)$
  end for 11. do     z = $h_{f}\left(t\right)\;\mathrm{tends}\;\mathrm{to}\;1$ and $h_{b}\left(t\right)\;\mathrm{tends}\;\mathrm{to}\;1$
    Concurrent activity detect   end For 12. do for t=0 to n, k = 0 to n-1 do $\Phi (z_{t},z_{t-1},y,t)=\exp\underset{k}{\sum }\lambda_{k}f_{k}(z_{t},z_{t-1},y,t),\Psi (z_{u},z_{v},y,u,v)=\exp\underset{k}{\sum }\lambda_{k}^{\prime }f_{k}^{\prime }(z_{u},z_{v},y,u,v)$
                   $\frac{\partial P\left(z|y\right)}{\partial \lambda_{k}}=\frac{\partial L}{\partial \lambda_{k}}=\frac{\lambda_{k}}{\sigma^{2}}$
        end for 13. Interleaved activity detect 14. end for 15. end for 16. end

## 4. Experimental Configuration

Our primary target is to recognize the complex activity using our novel approach. We experiment, evaluate, and analyze our proposed method using publically available datasets named; Kasteren House- B from Kasteren [38] and Kyoto 3 from CASAS [39]. The pseudocode for the proposed approach is shown in Algorithm 1. Then we compare the results with other HAR methods.

### 4.1. Benchmark Datasets

**Figure 8:**
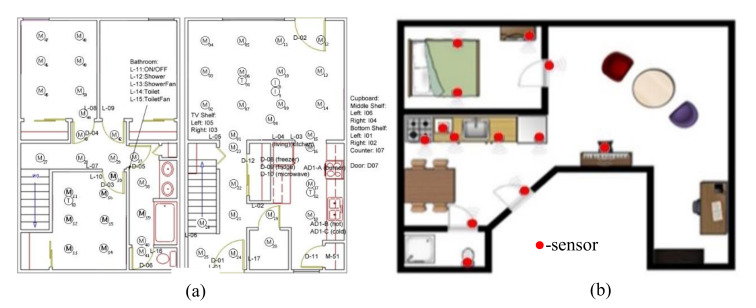
Sensor layout for (**a**) Kyoto 3 Dataset (**b**) Kasteren House B Dataset.

Human activities are unpredictable and periodic. There are numerous datasets available. Mostly, concerns with simple activity with low instances. Furthermore, the sensors used are body-worn. The proposed method focused on using external sensors rather than body-worn. Human activities offered a large Null class that is more challenging to identify the start and endpoint of data and classify it. In more recognition, we ignore the Null class, as including the null class may lead to an overestimation of the recognition. Table 1 shows an overview of the datasets. The chosen datasets count the limitation of sharing features among the activity instances of different groups, the number of inhabitants in each home, their performance of the same activity in different ways, and the availability of fewer data for learning. The twenty-three and seventy-six different heterogeneous sensors collect the data in Kasteren house-B and Kyoto, respectively. Among 76 sensors, 51 sensors were used to detect motion sensors and five sensors to detect the cooking item. Twelve sensors as a cabinet sensor, three sensors as a temperature sensor, and another sensor as a medicine container sensor, pot sensor, phone book sensor, water sensor, burner sensor, and phone sensor. For the Kasteren House, sensors like RFID (Radio Frequency Identification), Pressure sensor, mercury contacts, passive infrared-PIR, float sensors, reed switches, and temperature sensors are deployed to capture the data. The schematic sensor layout of the dataset is shown in Figure 8. There are 135 and 178 activity instances from which the 13 and 8 activity will be recognized in a concurrent and interleaved fashion. The sensors represent the change of state according to the action of users.

### 4.2. Parameter Setup and Training

**Figure 9:**
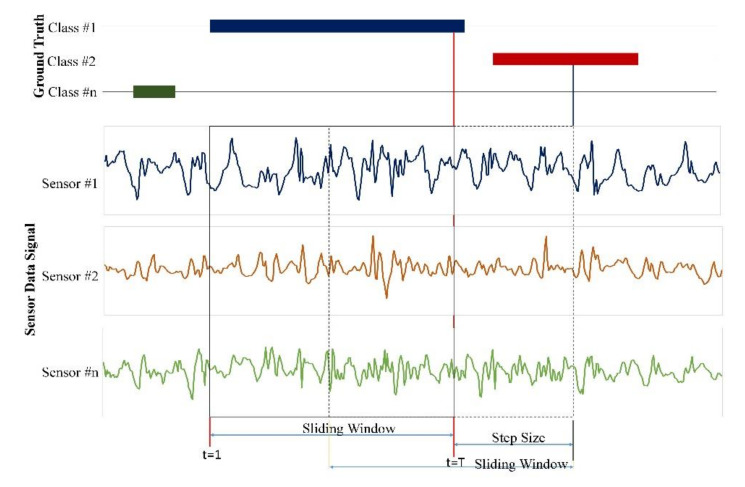
Sequence labeling after segmentation of data with a sliding window.

The datasets were segmented into different window ranges, as shown in Figure 9. The maximum ranges of windows depending on the activities performed by the human and the sampling rate. In this experiment, we choose the best ranges window that gave better results by performing various samples. In the uncertain and real-time scenario, varied window length plays a perfect role rather than fixed scales because the network has to wait to complete a given range to proceed further. When the mean cross-entropy of actual outputs and predicted outputs are measured as updated cost function, then a cost function must be minimized by randomly initializing the approach’s weight parameters. The actual output is given in the datasets and indicates real output activity for segmented windows. The predicted outputs contain every output generated by our method.

During the training iteration, the dropping at nodes in the network is tuned and represents units of the drop by using hyper-parameter. This dropping technique narrowed the accuracy gap between training and testing costs, which means dropping technique over through overfitting problems. The main reason behind using dropout is to disable the neurons with low probability, which improves the model generalization. The proposed method is implemented in the TensorFlow_gpu1.13.1 library. The computer taken for testing is best suitable to run our algorithm with i7 CPU with 16GB RAM with GTX titan GPU with CUDA 9.0 and used the cuDDN 7.0 library. CUDA is a platform that eases for parallel programming and also an interface for application programming. CuDDN is a GPU facilitated library of primitives for deep neural networks. 80% of the data are the training set, and the remaining 20% of data is for the test set. We record the Kasteren and Kyoto datasets. The training and testing use a sliding window of fixed length to segment the data, but data inputs may be variable length windows in real-time data scenarios. Following the preferred dataset experimental setup, the proposed approach depicts 500 ms lengthen window size with a 250 ms step size. The number of instances acquired after using this sliding window arrangement per the depicted dataset. The class corresponds to the observed sensor during that interval is associated with each segment. The sequence’s activity as the label at t = n, i.e., the last sample’s label in the sliding window of length ‘n’. A sliding window segments the sensor signals. The activity class within each activity sequence is the ground truth label annotated at the sample n of that window.

The best-suited hyper-parameters are tuned, as shown in Table 2, and achieve the possible optimal performance. We train the model with a learning rate of 0.005, and 100 batches of each epoch for the Kasteren House and the learning starts at 0.001. The training is carried out for more than 10000 epochs and stops on stable outputs. The Adam optimizer is an adaptive moment estimation that obtains independent adaptive learning rates for different parameters making them more stable for the Bi-LSTM and Quasi-Newton optimizer by the SCCRF. The dimension based on the input is set to 128. The gradient clipping is set to 5, whereas this parameter scaled-down the exceed gradient threshold to match the normalization. Also, In this paper, the SCCRF model uses the Limited-Memory Broyden-Fletcher-Gold-Shano (L-BFGS) algorithm to estimate the parameter, and the Loopy Belief Propagation (LBP) algorithm to perform the approximate inference. We used the 10-fold cross-validation technique to evaluate models. This may also help to estimate the capabilities of a model on unseen data.

### 4.3. Analysis Metrics

We define the evaluation terminologies as precision, recall, F-score, and accuracy. F-score is the harmonic mean of precision and recall. These terminologies are measured using true positive (TP), false positive (FP), and false-negative (FN) through the confusion matrix.

(15) $$Precision\;:\frac{1}{N}\;\left(\overset{N}{\underset{k=1}{\sum }}\frac{TP_{k}}{TP_{k}+FP_{k}}\times 100\right)$$

(16) $$Recall\;:\frac{1}{N}\;\left(\overset{N}{\underset{k=1}{\sum }}\frac{TP_{k}}{TP_{k}+FN_{k}}\times 100\right)$$

(17) $$F-score\;:\frac{2\times Precision\times Recall}{Precision+Recall}$$

(18) $$Accuracy=\frac{tp+tn}{tp+tn+fp+fn}\times 100$$

Each dataset is categorized into three-set: a training set, a validation set for optimizing the parameters, and a test set for end evaluation.

## 5. Activity Recognition Performance Analysis

In this section, the experimental results are explained and analyzed. All activities are located based on the dataset. The most frequently concurrent and interleaved activities are considered the presiding activities in the smart home environment. The analysis section is subdivided into concurrent activity recognition, interleaved activity recognition, and the recognition average. The overall recognition accuracy is then compared to the other state of art method; Hidden Markov model-Skip Chain Conditional Random field (HMM-SCCRF) [7], Long-Short Term Memory- Skip Chain Conditional Random field (LSTM-SCCRF) [40], and Bi-directional Long-Short Term Memory- Conditional Random field (Bi-LSTM-CRF) [41].

### 5.1. Concurrent Activity Recognition Analysis

**Figure 10:**
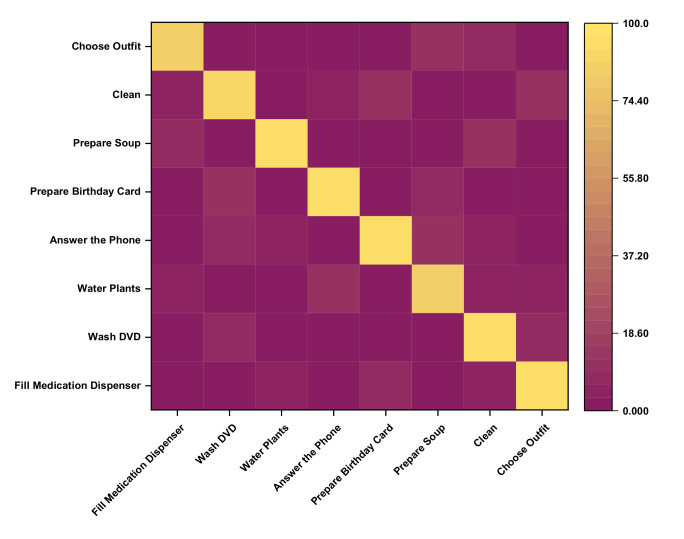
Confusion matrix for Kyoto 3.

**Figure 11:**
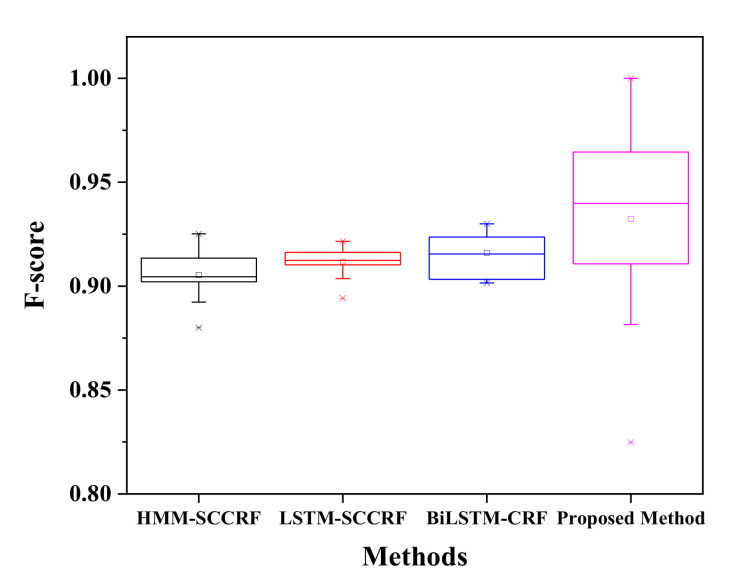
F-score comparison on Kyoto.

Figure 10 represents a confusion matrix for Kyoto 3 for the recognition of concurrent activities. The proposed approach recognizes activities with satisfactory accuracy of more than 90%. The filling medication dispenser and answering phone are concurrent with each other with an accuracy of 96% as both activity can be performed in parallel. Wash DVD, clean have 2% of their instances is shared as these activities share the same location. 3% of watering plants is due to some kinds of error factors such as placement of motion sensor, frequent movement of inhabitants, and confusion in the kitchen location. The watering can be retrieved from the kitchen supply closet. Answering the phone is recognized concurrently with all the other activity with an accuracy of 94%, 97%, 94%, 93%, 95%, and 95% to wash DVD, water plants, prepare birthday cards, prepare soup, clean, and choose outfit respectively. All the activities can be performed in a parallel manner while talking with high accuracy with ease. The Kyoto 3 data set’s overall accuracy in recognizing the concurrent activities is more than 95%, as shown in Figure 11, compared to other previously proposed approaches. There occurred a confusion between different activities trying to appear parallel with each other because of sharing the same location, platform, and some of the shared sensor values.

**Figure 12:**
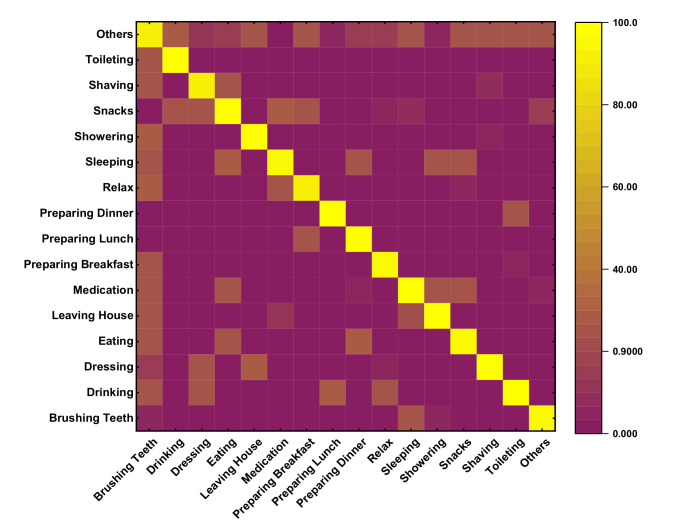
Confusion matrix for Kasteren-House-B.

**Figure 13:**
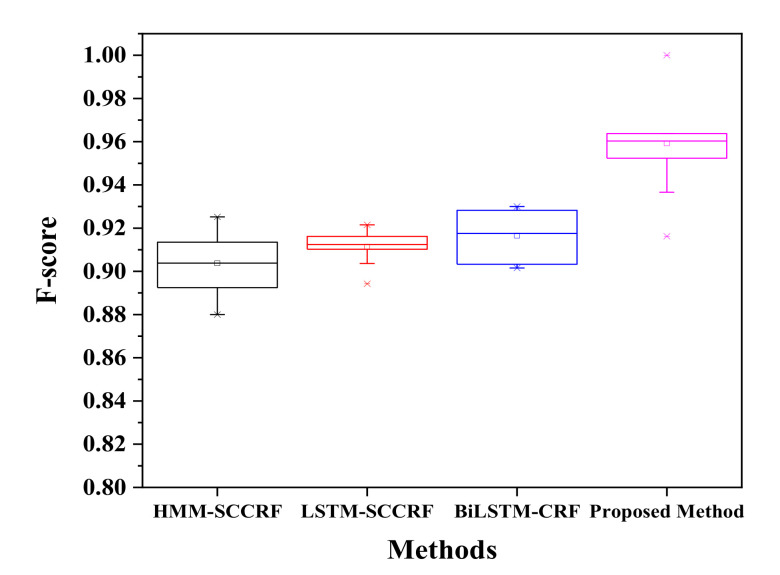
F-score comparison on KH-B.

On the other hand, Figure 12 represents a confusion matrix of Kasteren-House-B to recognize the concurrent activity. While preparing breakfast, drinking, and eating can be done at the same time. The preparing breakfast activity is recognized concurrently to drinking activity with an accuracy of 97%, and the same way, eating and drinking activities are detected concurrently with an accuracy of 93%. 10% of error for dishwasher activity, 4% of error for other activity creates confusion. These activities are carried out in the same location at the kitchen. During this session location sensors, they are common and activated simultaneously time. Drinking activity has a high percentage of appearing B with an average accuracy of more than 93% to concurrently with other activities like breakfast 88%, dinner 98%, leaving the house 75%. The only other activity does not have the concurrency because this activity is not well defined, and this is to reduce the error while recognizing the activity. The confusion matrix of two renowned datasets shows that recognizing concurrent activity using the proposed approaches and algorithm is quite impressive. However, a large number of datasets could help to get the actual and real recognition distribution. Kasteren and CASAS dataset have only a limited number of data and instances; therefore, it is a little bit easy to recognize, and accuracy may be high enough. Regarding analysis, our proposed method seems to be more reliable and effective than other compared models, as shown in Figure 13 on the same Kasteren house datasets.

### 5.2. Interleaved Activity Recognition Analysis

**Figure 14:**
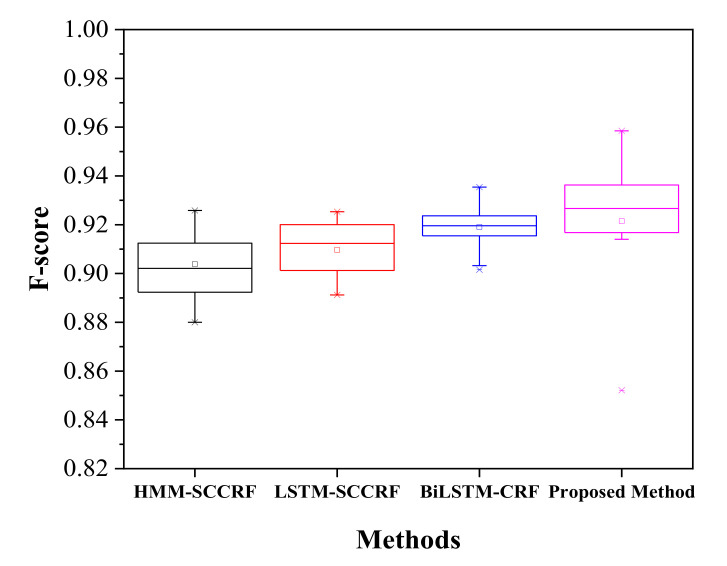
F-score comparison on KH-B.

Interleaving activity is more complicated and challenging to recognize than concurrent activities; here, the activity is detected again after it has been performed a time before and reappeared at a specific time paused. The confusion matrix for Kastern House-B to detect the interleaved activity is shown in Table 3. The chances of recognizing breakfast activity after being paused are 93% while it has external instances error of 2%, 2.3%, and 2.7% of dinner, breakfast, and others. Brushing Tooth can be re-recognized to 95%, with an error of 5.2% each to toileting and showering; this is because the given activities share some information like location and sensor values. Sleeping has a 97% chance to recognize interleave fashion, but it has 6% confusion to the brushing tooth. It is due to the simultaneous occurrence of brushing teeth activity and sleeping. Activities like Dinner, Drinking, Dressing, Leaving House, Preparing Breakfast, Preparing Dinner, Showering, Toileting, Using Dishwasher, Others have the accuracy of 91 %, 95%, 97%, 90%, 92%, 91%, 90%, 92%, 94% and 83% respectively. Therefore, the activity that appears other than the assigned activity is another activity. The proposed method recognizes the interleaving activities with an average accuracy of more than 92% with an f-score of 0.918, as shown in Figure 14. Although the proposed method shows good results of the recognition of interleaving activity, there are so many confusion values that occurred. The main reason for this type of confusion is all because of sharing common location, common sensor values, the simultaneous occurrences of activity, and sharing the same platform.

**Figure 15:**
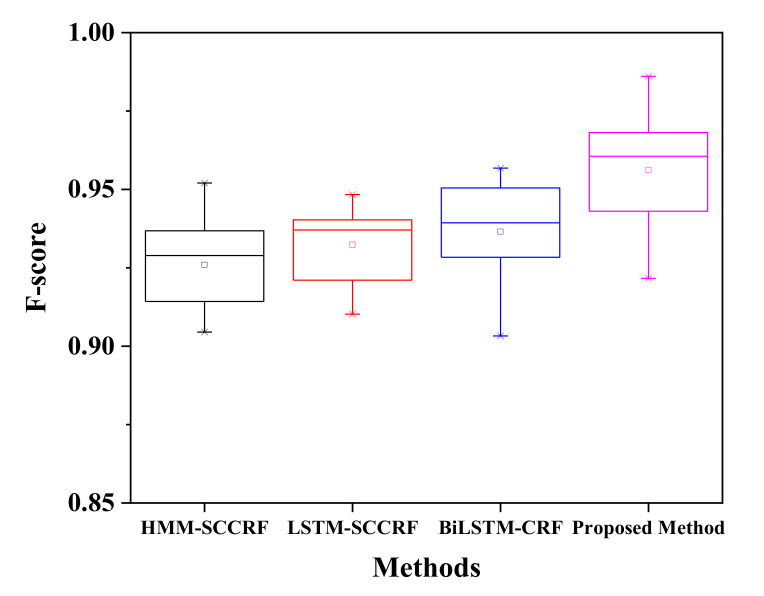
F-score comparison on Kyoto.

Table 4 represents the confusion matrix of recognition of interleaved activity for the Kyoto 3 dataset. The confusion matrix shows the average f-score of the recognition is above 0.95, as given in Figure 14, which shows the average accuracy is high to desire. As mention, due to limited data, the accuracy is pretty much in good shape. The recognition of the same activity, after pausing fill medication dispenser is about 93%. Recognition of interleaved activity like Wash DVD, Water Plants, Answer the Phone, Prepare Birthday Card, Prepare Soup, Clean, Choose outfit is satisfactory with an average accuracy of 90%, 92%, 90%, 91%, 91%, 90%, and 92% accordingly. The resultant recognition of interleaved activities by our proposed method outperforms existing approaches like HMM-SCCRF, LSTM-SCCRF, and Bi-LSTM-CRF. The resultant accuracy of Kyoto is more than 0.95 compared to other approaches, and algorithm 0.92 (HMM-SCCRF), 0.93 (LSTM-SCCRF), and 0.93 (Bi-LSTM-CRF) are as shown in Figure 15.

## 6. Conclusions

We adapt BiLSTM-SCCRF to establish a new approach to recognize concurrent and interleaved human activities within a single platform at a smart home. The proposed method recognizes activity in two phases. The first phase recognizes the concurrent activity using the bi-directional long short term memory (BiLSTM) extended version of RNN. The second phase is responsible for recognizing the interleaved activities using the skip-chain conditional random field (SCCRF) upgraded CRF version. By cascading these two phenomena, we can easily detect complex activity. This paper uses the two datasets to evaluate the proposed method’s recognition performance with other recognition methods. In the evaluation process, F-score and accuracy are calculated from precision and recall. Also, Table 5 represents the mean and standard deviation by varying the Batch size hyperparameter on 10,000 epochs. Similarly, Table 6 shows the epochs parameter’s result on sizing batch to 100, and the mean and standard deviation are calculated. The window size selection also plays a vital role in system accuracy; technically, 500 ms to 5000 ms window size extent will be relevant. The mean and standard deviation of accuracy and error using the 10-fold cross-validation is shown in Table 7. The average accuracy was found to be 92.69 ± 4.565%.

**Figure 16:**
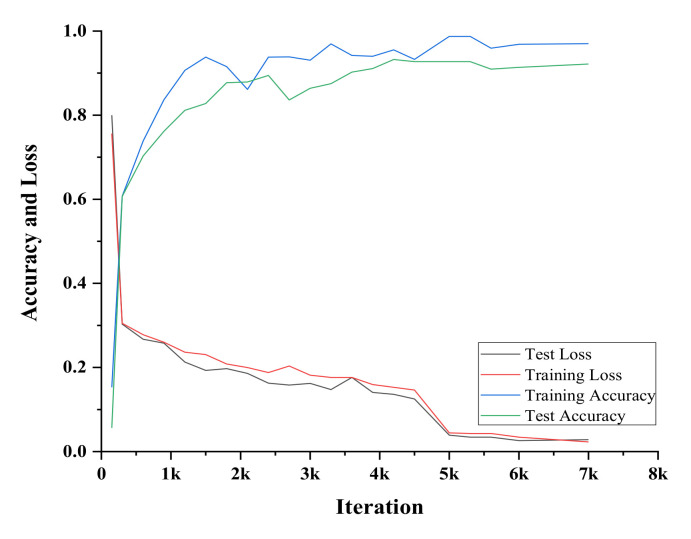
Accuracy of Training or Testing.

Figure 16 shows the training and testing accuracies of our method. The graph depicts successful training and recognition of activity in accordance. The training loss is comparatively more than test loss, which is natural when the training goes lots of phases to learn the activity’s actual occurrence.

Therefore, the proposed approach recognizes that complex activity, like concurrent and interleaved activity, is better than other techniques. The window size is also an important parameter to consider as too small size may not contain all information and too large causes the vacant producing recognition errors.

**Figure 17:**
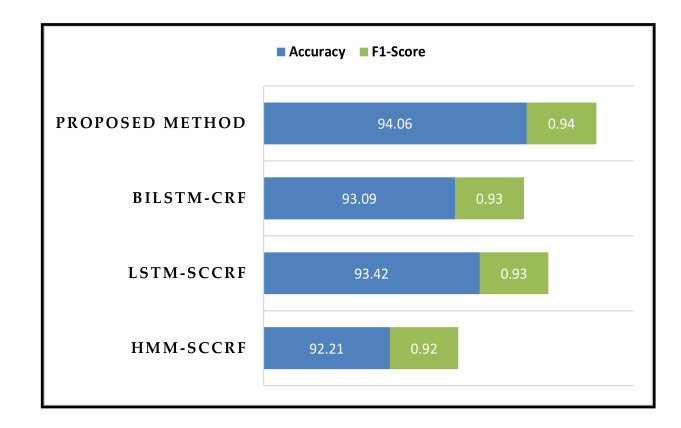
Overall accuracy and F-score of the proposed method.

Figure 17 shows the accuracy and the F-score of the proposed method compared to the other well-known approaches. Our proposed method outperforms other existing approaches holding accuracy of around 94% on average. Our proposed method’s distinguishing feature is that both the concurrent and interleaved activities are recognized through the single platform; instead, others use a different platform to recognize each activity separately.

We did our best to develop an adaptive way to adjust the searching process, reshaping, adding, and removing various layers. However, the excellent approach could be who has perfect generalization. The resident’s activities are recognized effectively and wisely by using the binary sensors deployed in smart homes. The external sensors have been used rather than wearable and camera or video sensors to avoid the burden and protect the inhabitant’s privacy. In the future, more complex activities like multiple users’ activity will be recognition by improving and updating the proposed method. As activity instances and training samples are not much, the technique could be suitable for many data like big data. We will also explore a transfer learning approach to this model to recognize the more complex activity on large-scale data and cloud infrastructures. In conclusion, it would seem preferable to use the model that offers fewer parameter complexities and computational loss.

## Figures and Tables

**Table 1:** Overview of datasets used in the evaluation of the proposed method.

Description	Kasteren House-B	Kyoto 3
Setting	Apartment	Apartment
Rooms	2	4
Senors	23	76
Activities	13	8
Residents	1	4
Period	14 d	15 d
Instances	135	178
Activities Performed	Breakfast, Brushing Teeth, Dinner, Drinking, Dressing, Leaving House, Others, Preparing Breakfast, Preparing Dinner, Sleeping, Showering, Toileting, Using Dishwasher	Fill Medication Dispenser, Wash DVD, Water Plants, Answer the Phone, Prepare Birthday Card, Prepare Soup, Clean, Choose Outfit

**Table 2:** Hyperparameter Settings.

Hyperprameters	Values
Time Steps of input	128
Dropout Rate	0.5
Initial Learning Rate	0.001
Learning Rates	0.005
Optimizer (Bi-LSTM)	Adam
Batch Size	100
Gradient Clipping	5
Skin-chain parameter θ	$\underset{k}{\sum }\lambda_{k}$
SC Optimizer	Quasi-Newton
Epochs	10000

**Table 3:** Confusion matrix for Kyoto 3.

	1	2	3	4	5	6	7	8	9	10	11	12	13	Recall
1. Breakfast	93	0	0	0	0.2	0.88	0	0	0	0	0	0	0	98.85
2. Brushing Teeth	0	95	0	0	0	0	0	0	6	0	0	0	0	94.06
3. Dinner	0	0	91	0	0	0	1.9	0	0	0	0	5.3	0	92.67
4. Drinking	0	0	0	95	0	0	0	4.6	0	0	0	0	2.3	93.23
5. Dressing	0	0	0	0	97	1.5	0	0	0	0	3	0	0	95.57
6. Leaving House	0.3	0	0	2.3	2.5	90	0	2.8	1	0	0	0	3	88.32
7. Preparing Breakfast	2	5.2	0	0	0	0	92	0	0	0	0	0	0	92.74
8. Preparing Dinner	0	0	0	0	0	0	0	91	0	2.7	0	1	0	96.09
9. Sleeping	0	5.2	0	0	0	0	1	0	97	0	0	0	0	93.99
10. Showering	0	0	0	0.2	0	0	0	0	0	90	1.6	0	0	98.04
11. Toileting	0	0	0	1.3	1.2	0	0	1.6	0	0	92	0	5	91.00
12. Using Dishwasher	0	0	7.2	0	0	0	0	0	0	0	0	94	0	92.89
13. Others	3	0	0	0	0	7.5	7	0	0	5	3	0	83	76.50
Precision	94.61	90.13	92.67	96.15	96.13	90.11	90.28	91.00	93.27	92.12	92.37	93.72	88.96	

**Table 4:** Confusion matrix for Kyoto 3.

	1	2	3	4	5	6	7	8	Recall
1. Fill Medical on Dispenser	93	1.3	2.7	0	0	1.6	0	0	94.32
2. Wash DVD	0	90	0	4	2	0	2	0	91.84
3. Water Plants	0	0	92	0	0	0	0	0	100.00
4. Answer the Phone	0.6	1.2	0	90	0	6	0	0	92.02
5. Prepare Birthday Card	0	3	0	0	91	0	0	2.3	94.50
6. Prepare Soup	5	0	0	2.3	3.2	91	0	0	89.66
7. Clean	2	0	5	0	1.2		90	1	90.73
8. Choose Outfit	0	4	0	0	0	2	2	92	92.00
Precision	92.45	90.45	92.28	93.46	93.43	90.46	95.74	96.54	

**Table 5:** Mean & standard deviation of variable range Batch Size on 10,000 epochs.

Batch Size	10	20	50	100
	Mean (μ) ± SD (σ)	Mean (μ) ± SD (σ)	Mean (μ) ± SD (σ)	Mean (μ) ± SD (σ)
House B	0.9261 ± 0.0734	0.9332 ± 0.0479	0.9327 ± 0.0413	0.9234 ± 0.0458
Kyoto	0.9407 ± 0.0620	0.9334 ± 0.0479	0.9419 ± 0.0413	0.9394 ± 0.0458

**Table 6:** Mean & Standard deviation of different Epcohs on 100 Batch Size.

Epochs	1000	5000	8000	10000
	Mean (μ) ± SD (σ)	Mean (μ) ± SD (σ)	Mean (μ) ± SD (σ)	Mean (μ) ± SD (σ)
House B	0.9030 ± 0.0655	0.930 ± 0.0468	0.9295 ± 0.0353	0.9103 ± 0.0482
Kyoto	0.9175 ± 0.0613	0.9303 ± 0.0347	0.9388 ± 0.0304	0.9411 ± 0.0464

**Table 7:** 10-fold Cross-validation Result.

	Mean ± SD Accuracy	Mean ± SD Error
House B	0.9148 ± 0.0458	0.476161 ± 0.15032
Kyoto	0.9390 ± 0.0455	0.31367 ± 0.21140
